# Who Needs a Friend? How Age and Having Someone one can Count on Explain Subjective Well-Being in India

**DOI:** 10.1093/geroni/igab046.3321

**Published:** 2021-12-17

**Authors:** Erika Sanborne

**Affiliations:** University of Minnesota--Twin Cities, West Saint Paul, Minnesota, United States

## Abstract

Subjective well-being is now considered a reliable predictor of many desirable outcomes not only at the individual level, in terms of one’s personal health, but also at the national level, in terms of a nation’s per capita gross domestic product, progress towards reaching sustainable development goals, and other metrics. Subjective well-being has several known causes that reliably predict well-being across time and place, such as income, education, prosociality, and perceived corruption (i.e. REF). Given the benefits of well-being to both individuals and nations, and that subjective well-being is often predicted by variables that are not easily altered, this study aims to better understand the relationships between subjective well-being and some of its known predictors, in the context of India. Three hypotheses were tested and found significant with nationally representative samples of a total of 57,077 survey respondents in India, using data from Gallup World Poll for 2006-2019. Hypothesis #1 tests for having someone one can count on as a mediator. Hypothesis #2 tests for age as a moderator. Hypothesis #3 is a moderated mediation that best explains how the known predictors of subjective well-being make their influence, and with whom. This study’s findings give insights into the ways in which subjective well-being in India can be better understood and thus improved. Such understanding may also help local Indian nonprofit organizations, as well as other researchers and mental health providers, with shared interests in the growing prevalence of suicide in India.

